# Epitopes in α8β1 and other RGD-binding integrins delineate classes of integrin-blocking antibodies and major binding loops in α subunits

**DOI:** 10.1038/srep13756

**Published:** 2015-09-09

**Authors:** Norihisa Nishimichi, Nagako Kawashima, Yasuyuki Yokosaki

**Affiliations:** 1 Cell-Matrix Frontier Laboratory, Health Administration Center, Hiroshima University, 1-2-3 Kasumi, Minamiku, Hiroshima 734-8551, Japan; 2 Clinical Genetics, Hiroshima University Hospital, 1-2-3 Kasumi, Minamiku, Hiroshima 734-8551, Japan

## Abstract

Identification of epitopes for integrin-blocking monoclonal antibodies (mAbs) has aided our understanding of structure-function relationship of integrins. We mapped epitopes of chicken anti-integrin-α8-subunit-blocking mAbs by mutational analyses, examining regions that harboured all mapped epitopes recognized by mAbs against other α-subunits in the RGD-binding-integrin subfamily. Six mAbs exhibited blocking function, and these mAbs recognized residues on the same W2:41-loop on the top-face of the β-propeller. Loop-tips sufficiently close to W2:41 (<25 Å) contained within a footprint of the mAbs were mutated, and the loop W3:34 on the bottom face was identified as an additional component of the epitope of one antibody, clone YZ5. Binding sequences on the two loops were conserved in virtually all mammals, and that on W3:34 was also conserved in chickens. These indicate 1) YZ5 binds both top and bottom loops, and the binding to W3:34 is by interactions to conserved residues between immunogen and host species, 2) five other blocking mAbs solely bind to W2:41 and 3) the α8 mAbs would cross-react with most mammals. Comparing with the mAbs against the other α-subunits of RGD-integrins, two classes were delineated; those binding to “W3:34 and an top-loop”, and “solely W2:41”, accounting for 82% of published RGD-integrin-mAbs.

Integrins are heterodimeric cell surface proteins expressed on virtually all cell types^1^. Non-leukocyte integrins function as primary receptors for extra-cellular matrix proteins^2^. The integrin family consists of 24 heterodimeric receptors composed of 18 α and 8 β subunits. Upon ligand engagement, conformational changes are relayed from the binding pocket to the cytoplasmic domains that are associated with adaptors and signalling molecules to initiate biochemical signals that modulate cell behaviour^3^. Inversely, cellular activation triggers changes from cytoplasmic to extracellular conformations to regulate ligand binding.

To identify critical amino acid residues in integrins for ligand-binding or other functions, a number of studies with site-directed mutagenesis of integrins or their ligands have been performed^4^,^5^. Three critical residues for ligand binding in the α5 subunit were identified by alanine mutagenesis^6^. Equivalently, epitope mapping for blocking monoclonal antibodies (mAb) against integrins have greatly contributed to defining key functional regions. For example, the binding site of the PHSRN fibronectin synergy peptide in α5β1 was localised within the α5 subunit, using two blocking mAbs, P1D6 and JBS5^7^, unlike the RGD-binding pocket that spans both α and β subunits. Identifying the epitopes of integrin blocking mAbs, sometimes in combination with the results of mutagenesis, has increased our understanding of the integrin structure-functional relationship^8^.

The N-terminal repeated sequence in the α subunit was found to form a β-propeller tertiary structure *in silico*^9^, which was subsequently confirmed by the solved crystal structure of αvβ3^10^ and αIIbβ3^11^. Key binding residues of previously identified blocking mAbs were each mapped onto three-dimensional (3-D) structures^11^,^12^ including homology-modelled α5β1^13^. These 3-D mappings showed a clustering of key amino acids for recognition within the same regions among RGD-binding integrin α subunits: three loops on the top face of the β-propeller^13^. Because the ligand-binding pocket of integrin is on the top face of the β-propeller^10^ and is close to these three loops, these blocking mAbs for RGD-integrin are likely to directly inhibit ligand binding.

Epitope mapping of integrin blocking mAb has been mostly performed by mutagenesis, which often identifies only a single loop as the epitope. In contrast, recent co-crystallisation and atomic coordination studies of integrin with blocking mAbs revealed that multiple loops serve as the epitope for a single mAb, of note that a loop on the bottom face of the β-propeller is an epitopes for two mAbs: 10E5 (αIIβ3)^11^ and 17E6 (αvβ3)^14^. The bottom loops have not been studied in detail. Therefore, whether binding to the bottom face is common to many blocking mAbs, and whether the bottom loop is specific is poorly understood. Bottom-binding mAbs, 10E5, 17E6 and mAb 16 bind to, αIIb, αv and α5^15^, respectively, three of four different α subunits in RGD integrins. Mapping of the epitopes for blocking antibodies against the only unstudied subunit of RGD binding integrins, α8^16^, would provide a more thorough understanding of the principals underlying antibody-mediated inhibition of the function of these integrins and might further aid our understanding of function of RGD-integrins.

There are many blocking antibodies to most integrin heterodimers, yet none have been reported that block α8β1, despite efforts to generate blocking antibodies by immunising mice, rat and rabbits (Personal communications from Lynn Schnapp, Medical University of South Carolina, and Dean Sheppard, UCSF). The α8 subunit is expressed preferentially on mesenchymal cells but not on epithelial cells^17^. Integrin α8β1 is essential for kidney development^18^ and has a role in lung and liver fibrosis^19^, and smooth muscle function^20^,^21^,^22^. An obvious limitation of these functional studies on α8β1 is the lack of a blocking mAb.

To address the absence of critical reagents to study α8β1, we obtained anti-α8 blocking mAbs in chickens. We reasoned that considering the phylogenic distance, the avian host might respond to divergent sequences in the antigen that are shared among mammals. We identified key recognition residues for anti-α8 blocking mAbs, and localised them to two loops. Taken together with the known epitopes, we delineated two classes of mAbs and two critical blocking antibody binding sites in RGD-integrins. The amino acid sequences in the α8 binding sites were remarkably conserved across virtually all mammalian species, which probably explains the failure of previous efforts to generate such antibodies by immunizing mammals.

## Results

### Classification and characterisation of integrin α8 mAbs

**Figure 1:**
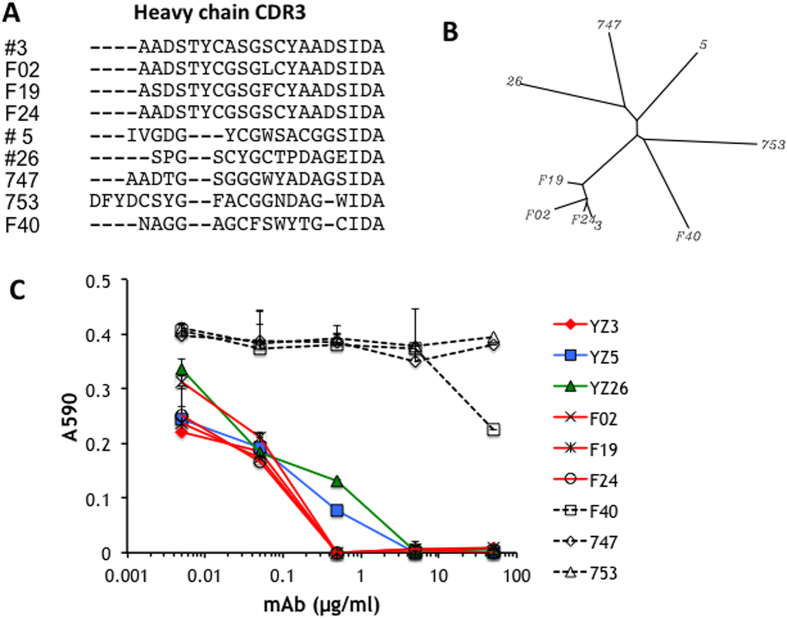
Characterisation of nine anti-human α8 mAbs. (**A**) Alignment of CDR3 amino acid heavy chain sequences. Clone names are shown on the left. (**B**) Phylogenic tree of the heavy chain CDR3 sequences by ClustalW. (**C**) Blocking activities of anti-α8 mAbs assessed by cell an adhesion assay using α8-transfected K562 cells and recombinant nephronectin with 0.005–50 μg/ml of each mAb. The red bar represents YZ3 and its three related clones. The three dashed lines represent non-blocking mAbs. Each dot represents the mean value of triplicate wells and lines above each dot represent the standard deviation.

We generated recombinant mAbs against the integrin α8 subunit in chickens to obtain functional blocking reagents for α8β1. Chickens were immunised and screened with mouse α8, which resulted in 10 positive clones that recognised mouse α8 expressed in SW480 cells. Controls consisted of mock-transfected SW480 cells that were not recognised by any mAbs. Overall, 9 of the 10 positive clones also reacted to human α8, and these were subjected to the following experiments. To exclude the possibility of identical clones, sequences in the complementarity determining region (CDR) of each positive clone were first determined and aligned (Fig. 1A). Each of the antibody sequences was unique. However, there was a marked similarity among four clones, #3, F02, F19 and F24. A phylogenic tree of the CDR sequences drawn by ClustalW (Fig. 1B) indicated the four clones were in the same group. We next assessed the blocking activity of each of the nine mAbs by performing cell adhesion assays with a recombinant fragment of nephronectin^23^,^24^ (Fig. 1C). Six of the nine clones (#5, #26 and the four related clones, #3, F02, F19 and F24) completely blocked cell adhesion at a concentration of 5 μg/ml. Clones 747, 753 and F40 did not inhibit adhesion at concentrations up to 50 μg/ml, and these were classified as non-blocking antibodies. Because of the high CDR sequence similarity among clones #3, F02, F19 and F24 and that they all had a similar blocking potency, we considered they might recognise a closely related epitope and used clone #3 for all subsequent studies. The three blocking clones, #3, #5 and #26 were termed mAbs YZ3, YZ5 and YZ26, respectively.

### Epitope mapping based on homology

**Figure 2:**
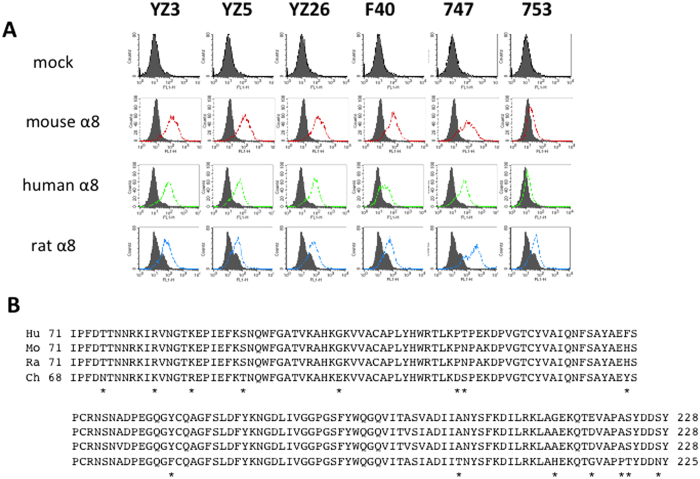
Cross-reactivity of mAbs to human, mouse and rat α8. (**A**) CHO cells expressing human, mouse or rat α8 were subjected to FACS with anti-α8 mAbs. (**B**) Sequences of putative integrin α subunit epitope regions from human, mouse or rat α8 are aligned with chicken α8. Asterisks (*) indicate 15 positions where the chicken residue has a variant compared with human, mouse or rat α8.

**Figure 3:**
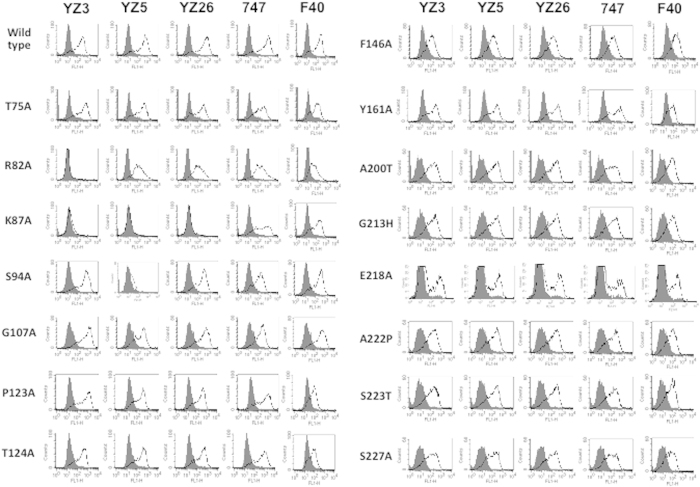
Recognition of anti-α8 mAbs for mutant α8 by FACS. FACS-analyses of 15 CHO cell-lines each stably transfected with mutant human α8 cDNA in which a single non-conserved residue from chicken shown to the left has been mutated.

The phylogenic distance of chicken from mammals and an excessive cross-reactivity ratio of the obtained anti-mouse clones to humans (90%), suggested that chicken-derived mAbs would have a wide reactive spectrum for mammals. We next examined reactivity of the mAbs to rat α8. As expected, all six mAbs recognised also rat α8 equally well to mouse and or human α8 by flow fluorescence-activated cell sorting (FACS) (Fig. 2A). Because none of the mAbs recognised mock-transfectants, the shift in the histograms demonstrated recognition of the transfected α8 expression. In addition, each of fluorescent intensities of mock-transfectants stained with the anti-α8 mAbs as primary antibodies was the same as that stained without the primary antibody, indicating minimal nonspecific binding of the anti-α8 mAbs. The cross-species reactivity of the six mAbs across mouse, rat and human indicated the α8 protein of humans, mice and rats should share the same or highly homologous sequences that are different from chicken α8. To identify epitopes of the blocking YZ3, YZ5 and YZ26 mAbs, we aligned the chicken α8 sequence with mouse, rat and human α8 on epitope-clustering blades two to three of the β-propeller domain^12^ (Fig. 2B). There were 15 positions where the chicken residue was different from the three other species. In 11 of these 15 positions, the human, mouse and rat sequences were identical. We performed mutagenesis for each of the 15 residues and analysed mAb recognition using CHO cell lines expressing each mutant. The 15 human residues were replaced with Ala unless any of the human, mouse or rat residues was Ala, in which case it was replaced with the corresponding chicken residue: Ala200Thr, Gly213His, or Ala222Pro (Fig. 3). Only three mutations affected the recognition of transfectants. First, all three blocking mAbs, YZ3, YZ5 and YZ26 lost recognition for α8 when Lys87 was mutated. Second, mAb YZ3 lost recognition for Arg82Ala. Third, YZ5 recognised Ser94Ala minimally. In sharp contrast, recognition of non-blocking mAbs 747 and F40 were not affected by any of the 15 mutations.

### The loop spanning W1 and W2 of the β-propeller (W2:41) is an α8 epitope

**Figure 4:**
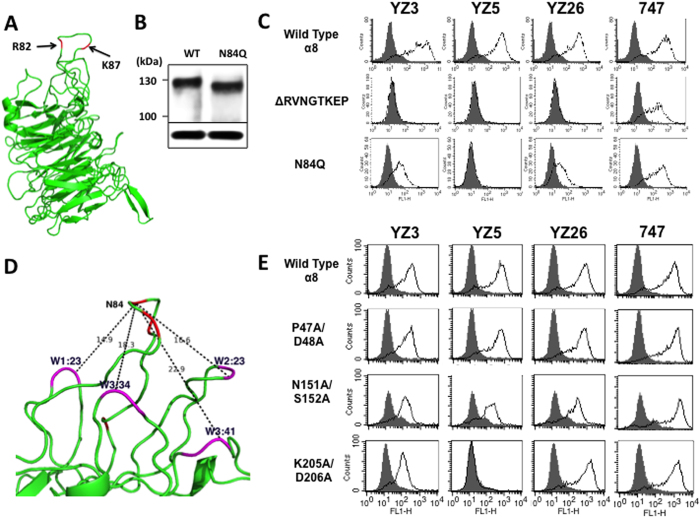
Anti-α8 mAbs recognise epitope loops, W2:41 and W3:34, in the β-propeller. (**A**) Localisation of Arg82, Lys87 and Ser94 key residues (arrows) in putative 3-D image homology-modelled with the αv structure. (**B**) Mobility shift of the Asn84Gln mutant in the 84Asn-Gly-Thr86 *N*-glycosylation sequence by western blotting. Bands at the bottom show GAPDH as a loading control. (**C**) Recognition of anti-α8 blocking mAbs for top region W2:41 (middle panel) deletion mutants and Asn84Gln mutants (bottom panel). 747 is a control non-blocking mAb. (**D**) Distances (Å) from the tip of W2:41 to the tip of the four closest loops are shown in a putative α8 3-D image. Red represents key residues including Asn84, and magenta represents two residues on the tip of each loop. (**E**) FACS-recognition for three mutants in which the tip-residues are replaced with Ala.

To determine the localisation of the key residues Arg82, Lys87 and Ser94 in α8 the residues were mapped onto a 3-D image generated as a homology-model through SWISS-MODEL^25^ web site using αv (3IJE) as a template. The three key residues were all located on the same loop, W2:41 (based on the first model of the α subunit structure^9^), two of which, Arg82 and Lys87, were near the tip of the loop (Fig. 4A). Between these two key residues, there was an *N*-glycosylation sequence, 84NGT86 (Asn-X-Thr/Ser)^26^, which led us to examine whether the binding site of YZ3, YZ5 or YZ26 included the *N*-glycan. An Asn84Gln mutant α8 was generated to abolish the *N*-glycan and was expressed on CHO cells. Because the *N*-glycosylation sequence is not sufficient for glycosylation^26^, we first assessed the mobility shift between wild type and Asn84Gln mutant α8 by SDS-PAGE to confirm the presence of the *N*-glycan (Fig. 4B). The reduction of the molecular mass in the Asn84Gln mutant was compatible with the removal of the *N*-glycan, indicating its existence (Fig. 4A). FACS analyses demonstrated that recognition by YZ3 and YZ26 was attenuated and that of YZ5 was completely lost by the mutation (Fig. 4C), while mAb 747 showed the same intensity between wild type and the mutant. Thus, all three mAbs included the *N*-glycan in their epitopes, especially YZ5, where the *N*-glycan is critical for binding to α8.

To exclude the possibility that mutation-induced allosteric conformational changes affected the loss of recognition, we mutationally deleted the W2.41 loop. Stably transfected CHO cells expressing the mutant lacking 82RVNGTKEP89 were not recognised by the three blocking mAbs, but binding of the non-blocking mAb 747 was unaffected (Fig. 4C). These results confirmed that the W2:41 loop is crucial for the recognition of all the blocking mAbs.

### Epitope mapping based on topology

A loop on the lower face of the β-propeller, W3:41, was reported to be an interaction site of two integrin blocking mAbs, 10E5 (αIIb)^11^ and 17E6 (αv)^14^. The interactions with W3:41 were determined using atomic co-ordination of crystal structures by computer analyses, which determined the human residues were identical to mice residues and were critical for both interactions. Because both 10E5 and 17E6 bound to multiple loops including W3:34, this indicated that similar to these mAbs, the α8 mAbs might have more binding loops regardless of amino acid conservation. If additional loops are present, the distance from W2:41 should be within a foot print of the mAb, which extends over a large area of 30 × 20 Å^27^,^28^. We examined the 3-D image of α8 to find additional binding loops that were sufficiently close to W2:41 to be shared in the same epitope. Although the distance deduced from the 3-D modelled image may not be accurate, there were four loops in which the tips were less than 25 Å from the tip of W2:41 (Fig. 4D). Amino acids on the tips of these loops, W1:23, W3:41 and W3:34, were mutated except for W2.23, because Pro123A and Thr124A mutations on W2.23 were initially performed as two of the 15 non-conserved residues in chickens (Fig. 2B) and were determined not to be a binding site. FACS analysis demonstrated that mAb YZ5 did not recognise the mutant Lys205Ala/Asp206Ala on W3:34, while YZ3 and YZ26 recognised it with the same intensity as wild type α8. The two mutations on W1.23 and W3:41 did not affect the recognition of any of the three mAbs (Fig. 4E). Control mAb 747 showed that each of the three mutants was expressed equally compared with wild type α8. Thus, Lys205/Asp206 on W3:34 were key residues for the binding of YZ5. Of note, Lys205/Asp206 on W3:34 was conserved in chicken α8.

### Epitopes of non-blocking mAbs

**Figure 5:**
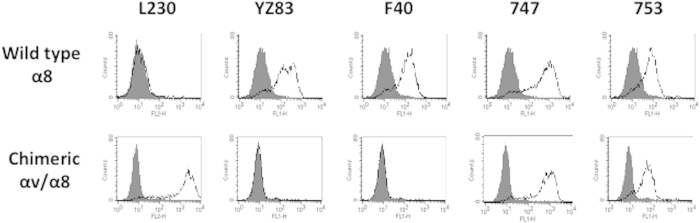
Epitopes recognised by non-blocking mAbs. FACS-analyses with α8 non-blocking mAbs, F40, 747 and 753 for wild type and mutant α8 in which the β-propeller domain is replaced with that of αv subunit (αv/α8). L230 and YZ3 recognise the β-propeller domain of αv and α8, respectively, and were used to demonstrate the expression of αv/α8.

We identified eight key amino acid residues for binding of the three blocking mAbs. All residues were localised at loops W2.41 or W3.34. Next, we localised the epitopes of the three non-blocking mAbs and determined whether they were located in the β-propeller. The entire β-propeller domain of human integrin α8 was replaced with that of αv by connecting Arg438 of αv to Pro447 of α8. The chimeric αv/α8 protein was expressed in CHO cells and the transfected cells were analysed by FACS (Fig. 5). Anti-αv blocking mAb L230 (Tetsuji Kamata, Keio University, and Yasuyuki Yokosaki, unpublished observation) and anti-α8 YZ3 both specifically recognised the β-propeller domains. As expected, L230 did not recognise α8-expressing cells, but did recognise αv/α8-chimeric α subunit expressing cells. Anti-α8 YZ3 recognised α8-expressing cells but did not bind to αv/α8-expressing cells. These results indicated that the chimeric αv/α8 was correctly expressed, and that L230 did not recognise hamster αv and wild type α8 that was not expressed on the surface of these cells. The non-blocking mAbs, 747 and 753, recognised both wild-type α8 and αv/α8 expressing cells, indicating that both mAb 747 and 753 recognise the α8 extracellular domain in a region other than the β-propeller. In contrast, non-blocking mAb F40 did not recognise αv/α8-cells but did recognise wild type α8-cells, suggesting that F40 bound within the β-propeller domain; however, because F40 did not bind to W2 or W3 of the β-propeller domain (Fig. 3), F40 appeared to bind to anywhere in W1, W4, W5, W6, and W7.

### Conservation of α8 epitopes across mammals

**Figure 6:**
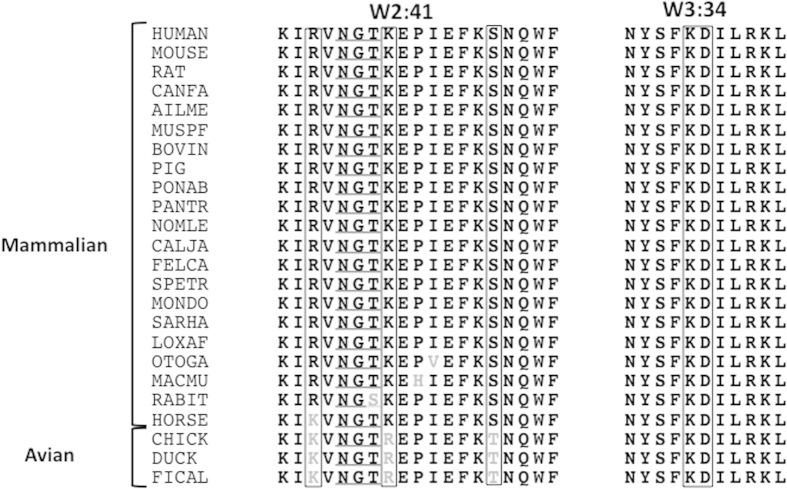
Alignments of α8 epitope sequences for mammals. The human KIRVNGTKEPIEFKSNQWF sequence including R82, K87 and Ser94 key residues and 84NGT86 *N*-glycosylation sequence, and NYSFKDILR including K205/D206 key residues are aligned with 20 mammalian and three avian sequences including chicken. Non-conserved residues are shown in grey. Human key residues and the corresponding residues in other species are shown in boxes. *N*-glycosylation sequences are underlined. Mnemonic organic identification codes of the UniProtKB data base are shown to the left of the sequences: CANFA represents *Canis familiaris* (Dog), UniProtKB ID of the source protein is F1PVR1; AILME, *Ailuropoda melanoleuca* (Giant panda), G1L8J0; MUSPF, *Mustela putorius four* (European domestic ferret), M3YJ77; PONAB, *Pongo abelii* (Sumatran orangutan), H2N9U6; PANTR, *Pan troglodyte*s (Chimpanzee), H2Q1N7; NOMLE, *Nomascus leucogenys* (Northern white-cheeked gibbon), G1RP84; CALJA, *Callithrix jacchus* (White-tufted-ear marmoset), F7ALM4; MACMU, *Macaca mulatta* (Rhesus macaque), F7HGW0; OTOGA, *Otolemur garnettii* (Small-eared galago), H0WXM0; FELCA, *Felis catus* (Cat), M3VY58; SPETR, *Spermophilus tridecemlineatus* (Thirteen-lined ground squirrel), I3MA05; MONDO, *Monodelphis domestica* (Grey short-tailed opossum), F6TCV7; SARHA, *Sarcophilus harrisii* (Tasmanian devil), G3WIG1; LOXAF, *Loxodonta africana* (African elephant), G3TW88; FICAL, *Ficedula albicollis* (Collared flycatcher), U3JYV4; and TAEGU, *Taeniopygia guttata* (Zebra finch), H0YSX9. The human, mouse and chicken sequences were reviewed by UniProtKB.

α8 sequences, including the key residues on loops W2:41 and W3:34 (80KIRVNGTKEPIEFKSNQWF98 and 201NYSFKDILRKL211 in humans; key residues are underlined) were identical among humans, mice and rats as shown previously in the current study. Following the identification of key residues, we investigated more broad sequence conservation. Using the UniProtKB database, we compared all known mammalian sequences and three avian sequences (Fig. 6). The 201NYSFKDILRKL211 sequence was identical among all 21 mammals and three avian species evaluated. The 80KIRVNGTKEPIEFKSNQWF98 sequence showed nearly complete conservation among 21 mammals with four minor exceptions, including Arg82 to Lys in horses, and Thr86 to Ser in rabbits. Interestingly, the divergence in rabbits is within the *N*-glycosylation tripeptide and Ser in addition to Thr, is the only amino acid that supports *N*-glycosylation. In sharp contrast to the conservation of 80KIRVNGTKEPIEFKSNQWF98 across mammals, each of the key residues was divergent in chickens. These analytic results based on our epitope mapping suggest that the human W2:41 loop would be antigenic to chickens but not to mammals.

## Discussion

Using six blocking anti-α8 mAbs, we identified two binding sites including eight key residues based on the loss of FACS-recognition for Ala-replacement mutants. One binding site, determined by reference to its sequence variation, is located at the top of the β-propeller close to the ligand-binding pocket. The other binding site, determined by a topological approach, is on the tip of the loop on the bottom face of the β-propeller. The amino acid sequences in the binding sites are both surprisingly conserved across most mammals. The current study of α8 binding sites fulfils the epitope mapping of all four α subunits in the RGD-integrin subfamily. Lining up epitopes for αIIb, αv, α5 and α8 indicates two major classes of mAbs and defines the primary loop as an epitope in RGD-integrins. We use the terms ‘key residue’ based on the loss of FACS-recognition by mutation and ‘binding site’ as a region around the key residue, while ‘interaction’ includes those determined by atomic coordination based on a crystal structure.

**Figure 7:**
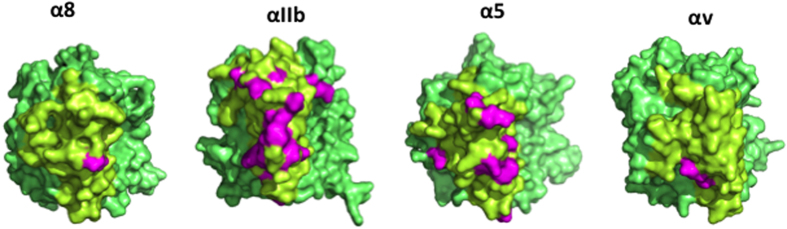
Sequence variations between mouse and human in α8 and the other α subunits in the RGD-binding integrin subfamily. (**A**) 3-D images of human integrin subunits α8 (homology-modelled with αv), αIIB (3FCU), α5 (3VI3) and αv (3IJE) on which non-conserved residues from mice are shown in magenta. The β-propeller domains of α subunits are shown in green and the putative epitope competent region, loops on the top face in W2 and W3, and loop W3:34 are shown in yellow.

The binding sites, loops W2:41^7^,^15^ and W3:34^11^,^14^, were previously reported as epitopes of blocking mAbs in other RGD-integrins. However, such extensive sequence conservation in epitopes across mammals has not been described for any integrin blocking mAb and the number of species cross-reactive to the mAbs might be the highest reported among integrin blocking mAbs. The long-term lack of an α8-blocking mAb generated in rodents or rabbits, in contrast to other α subunits, could be attributed to this conservation. To confirm the difficulty of producing an α8-blocking mAb, human non-conserved residues from mouse α8 were plotted on a 3-D image and compared with three other α subunits (Fig. 7). There are many variations in human αIIb and α5 compared with the mice equivalents, but only a few in αv and α8. In contrast to many mouse-generated blocking mAbs against αIIb and α5, few studies have described blocking mAbs for αv^29^ and none for α8. Since sequence divergence of immunogen are clearly important for generation, phylogenic distance is critical. Therefore using chickens to generate α8 blocking mAbs was successful.

The YZ5-binding to W3:34 loop supported the idea that a loop on the bottom face of the β-propeller serves as an epitope for some integrins. YZ5 also binds to the W2:41 loop on the top face through an interaction with the non-conserved residue Lys87, whereas peculiarly YZ5 in addition binds to the conserved residues with chickens, Lys205/Asp206 (Fig. 6) on W3:34. Why does YZ5 all the way binds to the bottom-loop W3:34 interacting even with conserved residues? One possibility is that the distance from W2:41 fits the foot print of YZ5 well. However, there are three other loops with a comparable distance to W2:41 (<25 Å), W1:23, W2:23 and W3:41 (Fig. 4D), and W2:23 in particular contains non-conserved, antigenic residues, which means W3:34 is not necessarily required for the binding site. Important conditions of B-cell epitopes were previously determined^30^. One critical condition of W3:34 is its accessibility. W3:34 turns at the bottom of the β-propeller toward the top face (Fig. 4A), and the tip of the loop is lateral to the β-propeller, which must be highly accessible, while access to the three other loops is easily hindered by the β-subunit or by loops on the α subunit. Thus, YZ5 binds loops on both the top and the bottom face of the β-propeller, and the key residues in the bottom loop W3:34 are conserved in chickens.

Because the last α subunit epitope of RGD-integrins has been mapped, we wanted to investigate whether the particular characteristics of anti-α8 YZ5 were distributed among blocking mAbs against other RGD-integrins, anti-αIIb, −αv and −α5, and whether there was any regulations common in RGD-integrins. We summarised the properties of these blocking mAbs (Table 1). We found that three blocking mAbs (10E5 (αIIb), 17E6 (αv) and mAb16 (α5)) had similar characteristics to YZ5: an interaction with W3:34 loop, interactions with top and bottom loops. The interaction with W3:34 is required for binding to α8 as confirmed by mutational experiments in αv, α5 and α8. For αIIb this has not been determined, but the residues of three (Arg208, Leu213 and His215) in the loop interact with Tyr residues in the 10E5 paratope. Therefore, the W3:34 in all RGD-integrins appears to function as a binding site. The binding of these mAbs to W3:34 is mediated by conserved residues with the host: Arg208, Leu213 and His215 in αIIb, Lys203 in αv and Ile210 in α5 are all identical to mice. The epitopes in α8 shares some characteristics with other α subunits in RGD integrins, which disclosed that there is a class of blocking mAbs that interacts with loops both on the top and the bottom faces, where the loop on the bottom face is specifically W3:34. Furthermore, each interaction with the loops is indispensable for binding, and the key residues in W3:34 are conserved in the host.

Apart from the structural demarcation, this lateral-binding class of mAbs appears to share other modes of action. mAbs in this class have been predicted to block by computerised docking analyses on crystal structures followed by inhibition experiments for two mAbs, 17E6 and Natalizumab^14^,^31^. The anti-α4 non-RGD-integrin mAb, Natalizumab, a humanised version of TY21.6 established in mice, also meets the above criteria. Although these mAbs block ligand-binding by steric hindrance similar to other blocking mAbs, their mode of action is unique. Docking analysis suggests that Natalizumab would not block domain 1 of VCAM-1, the integrin-binding domain in Ig-repeats, but blocks the immediately adjacent domain 2 from binding to the integrin. This is similarly observed for the inhibition on fibronectin-binding to αvβ3 by 17E6. The RGD-containing 10th domain of the type III repeat does not hinder 17E6 although the 9th domain does. These estimations were tested by binding-inhibition assays using ligand and cell lines, which showed indirect inhibition. Natalizumab is now available in the global market as a medical drug adapted for the treatment of multiple sclerosis^32^. 17E6 has been humanised and used in a clinical trial (EMD525797)^33^, which implies lateral-binding mAbs have a possible clinical advantage based on their pharmacokinetic characteristics.

Besides YZ5, all of our blocking mAb bound to loop W2:41 located on the top region of the β-propeller, where the β subunit or the β-propeller (Fig. 4A) does not appear to hinder mAb access. Table 1 shows seven mAbs that are not in the lateral-binding class, of which five recognise W2:41. This number is similar to that of the lateral binding class, and forms another class, “solely W2:41-binding”, including LJ-P9, JBS5, SNAKA5 and the three anti-α8 mAbs. Epitope mapping of these mAbs has been done for blades 1–3 of the β-propeller that covers the whole putative epitope competent regions but did not show epitopes other than W2:41. The two mAb classes account for 82% (9/11) of all blocking mAbs in Table 1. All epitopes were present within four loops, W2:41, W3:34, W3:31 or W2:23 with frequencies of 44%, 31%, 19% and 6%, respectively, in a total of 16 binding sites. In 11 mAbs with known epitopes, seven mAbs bound to W2:41. Taken together with its highly accessible location, W2:41 is the primary loop in the epitope recognised by blocking mAbs, at least in RGD integrins.

We confirmed the presence of *N*-glycan linked to 84NGT86, just within the epitope region recognised by the anti-α8 mAbs. YZ5 in particular required the *N*-glycan to bind to α8. The nearly complete conservation of the *N*-glycosylation NGT across all the mammals except rabbit, and the fact that NGS diverged only in rabbits indicates the necessity for *N*-glycosylation (Fig. 6), and suggests *N*-glycan should be critical for α8 functions. Indeed, a glycan on the β-propeller dramatically changes integrin basic functions including cell spreading and migration^34^. Although mAbs generated in rodents rarely recognise *N*-glycan^35^, our mAbs recognised the *N*-glycan, which may be explained by the divergence of glycans between mammals and chicken. In addition, the *N*-glycosylation on α8 W2:41 is the first description of a glycan within the epitopes of integrin blocking mAbs.

Because our 3-D image of α8 was a homology model based on αv, there is limitation in its accuracy. However, it was helpful in roughly estimating distances between loops, and led to the identification of Lys205/Asp206. Furthermore, although we collected all epitopes of blocking mAb reported from published data, there are many mAbs that still have unknown epitopes, and which are out of the scope of this study. However, the two major classes of mAbs are likely to be predominant because although total number of samples was small, they accounted for 82% of mAbs tested, and the binding sites in the two classes conform well the conditions that are used to determine B-cell epitopes.

Our results of anti-α8 mAbs together with previous epitope mapping studies allowed the classification of the blocking mAbs into two major classes, and defined the primary loop by epitope mapping. As Natalizumab, a non-RGD-integrin mAb, fulfils the conditions for the lateral-binding class based on RGD-integrins, the blocking mAb classification described here might be broadly applied to non-RGD-integrins such as α3, α4, α6, α7 and α9. Integrin blocking mAbs against 24 heterodimers are a class of biological agents that are expected to be useful once they have entered the drug market^36^. In conclusion, accumulated structural assessments for blocking mAbs help our understanding of the mechanisms of action of these mAbs, but only if their epitope has been determined.

## Methods

### Cells, Antibodies, and Reagents

Cell lines, Chinese hamster ovarian cancer CHO and human erythroleukaemia K562, were obtained from American Type Culture Collection (ATCC, Manassas, VA, USA). These cell lines were maintained in DMEM containing 10% foetal bovine serum, 100 U/ml penicillin, 100 μg/ml streptomycin and 250 ng/ml amphotericin B in 5% CO_2_ at 37 °C. FreeStyle 293-F cells were maintained in 293-F expression medium (Thermo Fisher Scientific, Waltham, MA, USA) in 5% CO_2_ at 37 °C. A hybridoma of the mouse anti-human integrin αv mAb L230 was obtained from ATCC. FITC-conjugated goat anti-chicken IgY was from Bethyl (Montgomery, TX, USA). Recombinant human nephronectin fragments corresponding to amino acids 378–403 were expressed as a GST fusion protein as previously described^37^. A retrovirus vector pMX was from Toshio Kitamura (The University of Tokyo, Japan)^38^.

### Antibody generation

Anti-α8 mAbs were generated by transfecting FreeStyle 293 F cells with chicken cDNAs encoding anti-α8 mAbs as previously described^39^. Briefly, chickens were immunised with a mouse-α8 expressing chicken T cell line, RP1^40^, then phage display libraries constructed from spleen mRNA of each chicken were screened by cell panning with SW480 human colon cancer cells transfected with mouse-α8 or recombinant soluble α8 protein. Then, mAbs were purified from the culture supernatant with nickel-nitrilotriacetic acid (Ni-NTA) agarose. The experiments were carried out in accordance with the Regulation of Animal Experiment of Hiroshima University, and all experimental protocols were approved by the Committee on Animal Experiment of Hiroshima University.

### Sequences of CDR in mAb

cDNA encoding CDR of the anti-α8 mAbs were sequenced by ABI Prism 3100. Ranges of CDRs in the mAbs were determined as described previously^41^.

### Mutagenesis

Site-directed mutagenesis was performed with the KOD Mutagenesis kit (Toyobo, Osaka, Japan) as described^42^. Then, the ligated PCR products were used for transformation of DH5α strain. The mutation in cDNA was verified before protein expression.

### Transfection

Mutant or WT α8 integrin subunit was expressed on CHO cells using retrovirus vectors. To obtain recombinant retrovirus, PLAT-GP cells were co-transfected with a pMX-neo retroviral vector containing the desired cDNA and envelope gene VSV-G. After 72 h of transfection, CHO cells were infected with PLAT-GP supernatant containing α8 retrovirus with 4 μg/ml hexadimethrine bromide, and then selected in culture for 10–14 days in the presence of 1 mg/ml G418.

### Cell adhesion assay

The adhesion assay was performed using K562 cells stably expressing human α8 integrin as described^43^. Cells were suspended in Tris-buffered saline containing 0.1% bovine serum albumin, 2 mM glucose and 1 mM MnCl_2_, then seeded onto 96-well plates coated with 2 μg/ml recombinant human nephronectin fragments in the presence or absence of anti-integrin mAbs and incubated for 1 h in 5% CO_2_ at 37 °C. The adhered cells were stained with 0.5% crystal violet, then solubilised with 2% Triton X-100 to take the optical density at 590 nm.

### Flow cytometry

After detachment from culture plates, cells were washed twice with phosphate buffered saline (PBS), incubated with primary antibodies in DMEM for 20 min on ice, and stained with secondary antibody, FITC-conjugated goat anti-chicken IgY (1:200) or PE-conjugated goat anti-mouse IgG (1:200). Data were acquired using a FACSCalibur flow cytometer and analysed using CellQuest Pro software.

### SDS-PAGE and western blot analysis

The cells were lysed for 30 min at 4 °C using Radio-Immunoprecipitation Assay buffer containing 1% aprotinin, 1% PMSF and a protease inhibitor cocktail. The lysate was centrifuged at 13,000 rpm for 15 min at 4 °C to remove the debris. After the protein concentration was determined, the samples were boiled for 5 min at 98 °C in SDS sample buffer, and resolved by 5–20% SDS-PAGE gel. For western blotting, the samples in the gel were transferred onto PVDF membranes which were blocked, then incubated with primary then secondary antibodies. Enhanced chemiluminescence emitted was detected and visualised using an imaging system.

## Additional Information

**How to cite this article**: Nishimichi, N. *et al.* Epitopes in α8β1 and other RGD-binding integrins delineate classes of integrin-blocking antibodies and major binding loops in α subunits. *Sci. Rep.* **5**, 13756; doi: 10.1038/srep13756 (2015).

## Figures and Tables

**Table 1:** Epitopes and characteristics of anti-human α subunit-blocking mAbs against RGD-integrin subfamily sequences.

Antibody	Host	Binding site	Key residues^a^ (human)	Glycan recognition	Reference
αIIb					
LJ-CP8	Mouse	W3:41	156VEND159	No	7
LJ-P9	Mouse	W2:41	79VGSQTL84	No	7
10E5^b^	Mouse	W2:41W3:34	77RNVGSQ82R208, L213, H215	No	11
αv					
17E6^c^	Mouse	W3:41W3:34	Q145K203	No	14
L230	Mouse	β-propeller	ND	ND	Unpublished
α5					
JBS5	Mouse	W2:41	S85	No	16
mAb16^d^	Mouse	W2:23W3:41W3:34	E126, L128S156, W157^44^I210^45^	No	16,44,45
P1D6^e^	Mouse	W3:34	Y208, I210, L212	No	16
SNAKA52	Mouse	W2:41	S85	No	16
α8					
YZ3 (and 3 related clones)	Chicken	W2:41	R82, K87	Yes (support binding)	This study
YZ5	Chicken	W2:41W3:34	84NGT86, K87, S94K205/D206	Yes (essential for binding)	This study
YZ26	Chicken	W2:41	K87	Yes (support binding)	This study

^a^Underline represents conserved residues with hosts.

^b^Determined according to atomic co-ordinations, and not confirmed by mutagenesis experiments.

^c^Determined according to atomic co-ordinations, and confirmed by mutagenesis experiments.

^d^mAb16 is suggested to have an additional binding site on the β subunit^46^.

^e^P1D6 blocks the synergy site but does not block binding of RGD-peptides to the binding pocket.
